# Regulation of ARHGAP19 in the endometrial epithelium: a possible role in the establishment of uterine receptivity

**DOI:** 10.1186/s12958-020-00689-7

**Published:** 2021-01-07

**Authors:** Jingjie Liang, Kui Li, Kaiyu Chen, Junyong Liang, Ti Qin, Jiayi He, Shuang Shi, Qiang Tan, Zhengguang Wang

**Affiliations:** 1 College of Animal Science, Zhejiang University, 866 Yuhangtang Road, 310058 Hangzhou, P. R. China; 2 Zhejiang Animal Husbandry Techniques Extension Station, 310020 Hangzhou, P. R. China; 3 Huzhou Southern Taihu Lake Modern Agricultural Technology Center, Zhejiang University, Huzhou, P. R. China

**Keywords:** ARHGAP19, Uterine receptivity, Epithelial transformation, miR-192-5p

## Abstract

**Background:**

The establishment of uterine receptivity is essential for embryo implantation initiation and involves a significant morphological transformation in the endometrial epithelial cells (EECs). The remodeling of junctional complexes and membrane-associated cytoskeleton is crucial for epithelial transformation. However, little is known about how this process is regulated in EECs during the receptive phase. ARHGAP19 is a Rho GTPase-activating protein that participates in various cytoskeletal-related events, including epithelial morphogenesis. Here, we investigated the role of ARHGAP19 in endometrial epithelial transformation during the establishment of uterine receptivity. The upstream regulator of ARHGAP19 was also investigated.

**Methods:**

ARHGAP19 expression was examined in mouse uteri during early pregnancy and in human EEC lines. The role of ARHGAP19 was investigated by manipulating its expression in EECs. The effect of ARHGAP19 on junctional proteins in EECs was examined by western blotting and immunofluorescence. The effect of ARHGAP19 on microvilli was examined by scanning electron microscopy. The upstream microRNA (miRNA) was predicted using online databases and validated by the dual-luciferase assay. The *in vivo* and *in vitro* effect of miRNA on endogenous ARHGAP19 was examined by uterine injection of miRNA agomirs and transfection of miRNA mimics or inhibitors.

**Results:**

ARHGAP19 was upregulated in the receptive mouse uteri and human EECs. Overexpression of ARHGAP19 in non-receptive EECs downregulated the expression of junctional proteins and resulted in their redistribution. Meanwhile, upregulating ARHGAP19 reorganized the cytoskeletal structure of EECs, leading to a decline of microvilli and changes in cell configuration. These changes weakened epithelial cell polarity and promoted the transition of non-receptive EECs to a receptive phenotype. Besides, miR-192-5p, a miRNA that plays a key role in maintaining epithelial properties, was validated as an upstream regulator of ARHGAP19.

**Conclusion:**

These results suggested that ARHGAP19 may contribute to the transition of EECs from a non-receptive to a receptive state by regulating the remodeling of junctional proteins and membrane-associated cytoskeleton.

## Background

The initiation of embryo implantation requires the differentiation of maternal endometrium into a special physiological state to accept embryo adhesion, which is called the receptive state [1]. During the transition from a non-receptive state to a receptive state, the components of the endometrium, including the epithelium and stroma, undergo significant changes. As the first contact between the maternal and embryonic tissue, luminal epithelium is thought to play a key role in the establishment of receptivity [2, 3]. Non-receptive endometrial epithelial cells (EECs) exhibit polarized characteristics of typical epithelial cells, with distinct apical and basolateral domains. Cells are tightly interconnected through multiple types of junctional complexes (such as tight junctions, adherens junctions, desmosomes) and form a two-dimensional sheet [4]. Meanwhile, the apical surface of non-receptive EECs is covered with actin-containing microvilli and does not exhibit adhesive properties [5]. These features of epithelial cells make the luminal epithelium a barrier for blastocyst adhesion and invasion. However, during the receptive phase, EECs experience a loss of polarity, and the cell-cell adhesion becomes weak. Moreover, the configuration of the cells transit from a columnar shape to a cuboidal shape, and microvilli on the surface retreat, which makes the apical membrane flat in favor of embryo attachment [2, 6]. Accumulating evidence suggests that the remodeling of junctional complexes and the reorganization of actin cytoskeleton are key factors that regulate morphological changes in cells, and cytoskeletal regulators, such as Rho-family GTPases as well as their regulators contribute significantly in this process [7, 8]. However, little is known about how these cytoskeleton regulators behave during epithelial transformation in establishing uterine receptivity.

The Rho family small GTPases serve as molecular switches that regulate multiple cellular functions, including various cytoskeleton-related events and gene transcription. The Rho GTPase-activating proteins (RhoGAPs) are one of the major classes of regulators of Rho GTPases found in all eukaryotes that are crucial in cell cytoskeletal organization, proliferation, differentiation, adhesion [8, 9]. ARHGAP19 is a member of the RhoGAP family and participates in the regulation of epithelial morphogenesis [10], whether it is involved in the epithelial transformation during the establishment of uterine receptivity remains unknown. In this study, we unraveled the expression pattern of ARHGAP19 in mouse uteri during early pregnancy and human EEC lines. Through manipulating ARHGAP19 expression in EECs, we showed that upregulating ARHGAP19 promotes the transition of EECs from a non-receptive phenotype to a receptive phenotype by regulating the remodeling of junctional complex and cytoskeletal structures. In addition, we also found that the expression of ARHGAP19 was regulated by miR-192-5p. These results suggest that ARHGAP19 may be involved in the establishment of endometrial receptivity by regulating epithelial morphology.

## Methods

### Animal treatments and ethical considerations

Healthy ICR mice were purchased from the Laboratory Animal Center of Zhejiang University (Hangzhou, Zhejiang, China) and housed in an environment with a controlled light cycle (12 h light/ 12 h darkness) and free access to food and water. Males (8 to 10-week old) and females (6 to 8-week old) were caged in the evening (6:00 p.m.) at a ratio of 1:1 to induce mating, and the morning of vaginal plug visualization was designated as day 1 of pregnancy (D1). Uteri from D1, D4, D5 were collected for RNA and protein evaluation. Implantation sites (IMS) on D5 were visualized by intravenous injection of 0.1 ml of 1% Chicago blue (Sigma, St Louis, MO, USA) [11], and the uterine tissue between two implantation sites was designated as inter-implantation sites (IIS). On the designated day, mice were euthanized through cervical dislocation [12] to collect uteri. For each experiment, uteri from three to five mice in the same group were set as biological replicates.

All protocols were approved by the Institutional Animal Care and Use Committee of Zhejiang University (ZJU20190151).

### Cell culture and transfection

The human endometrial adenosquamous carcinoma cell line HEC-1-A, Ishikawa, and the human embryo kidney (HEK) 293 T cell line (HEK293T) were purchased from the Cell Bank of Chinese Academy of Science (Shanghai, China) and cultured in plastic flasks with 5% CO_2_ in air at 37 °C. HEC-1-A cells were seeded in McCoy’s 5A medium (Invitrogen, Carlsbad, CA, USA) and Ishikawa and HEK293T cells were seeded in DMEM medium (Invitrogen). All the medium used were supplied with 10% FBS, 100 U penicillin, and 100 µg streptomycin (Invitrogen). Transfection of ARHGAP19 cDNA (2 µg), miR-192-5p mimics (50 nM, GenePharma, Shanghai, China), or inhibitors (100 nM, GenePharma) was performed using Lipofectamine 2000 (Invitrogen). Cells were collected 48 h after transfection for further study.

### *In vivo* injection of miRNA agomirs

Females on D3 of pregnancy (8:00 a.m.) were anesthetized and a surgery was performed to expose the uterus. 10 nmol/5 ul miR-192-5p agomir (GenePharma) was injected into one uterine horn, and the contralateral uterine horn was injected with an equal amount of scrambled control. Mice were sacrificed on D5 of pregnancy, and the uterine horns were collected separately for protein detection.

### RNA extraction and RT-qPCR

Total RNA was extracted from the uteri and endometrial cells using TRIzol (Invitrogen) according to manufacturer’s instructions. The quantity of RNA was examined using a NanoDrop2000 instrument (Thermo Fisher Scientific, Waltham, MA, USA). For mRNA detection, total RNA (2 µg) cDNA was synthesized using a FastKing gDNA Dispelling RT SuperMix kit (TIANGEN BIOTECH, Beijing, China). Gene expression was assessed by qPCR with 2 µl of the synthetized cDNA using a Real Universal Color PreMix (SYBR Green) kit (TIANGEN BIOTECH). For miRNA detection, total RNA (1 µg) was used to synthesize cDNA using a miRcute Plus miRNA First-Strand cDNA kit (TIANGEN BIOTECH) and miR-192-5p was qualified by qPCR using miRcute Plus miRNA qPCR (SYBR Green) kit (TIANGEN BIOTECH). The qPCR reactions were performed on a StepOnePlus™ Real-Time PCR System (Applied Biosystems Inc., Foster City, CA, USA). GAPDH/U6 were set as the normalizing control. Relative quantities were calculated using the 2^−△△CT^ method. The sequences of all primers used are listed in Table 1.

**Table 1:** Primer sequences for RT-qPCR

Gene symbol	Accession number	Primer Sequence (5’-3’)
U6	NR_004394.1	TTCGTGAAGCGTTCCATATTTT
miR-192-5p	NR_029720.1	CUGACCUAUGAAUUGACAGCC
GAPDH	NM_001256799	F: CTGGGCTACACTGAGCACC
		R: AAGTGGTCGTTGAGGGCAATG
Gapdh	NM_008084	F: AGGTCGGTGTGAACGGATTTG
		R: TGTAGACCATGTAGTTGAGGTCA
ARHGAP19	NM_032900	F: TGTGATCTGCAATGATTCTTCCC
		R: TTGCTGACCACCAACTCAGTG
Arhgap19	NM_001163495	F: CACAAGGCTTATTGATTTGCCG
		R: TTTCTTTCCGCTTGAGAGACATT
VIL1	NM_007127	F:GGCAAGAGGAACGTGGTAGC
		R:CGGTCCATTCCACTGGATGA

### Western blot analysis

Protein lysates were derived from tissues and cultured cells using RIPA buffer supplemented with 1 mM phenylmethylsulfonyl fluoride (Beyotime Biotechnology, Shanghai, China). The protein concentrations were detected using an Enhanced BCA Protein Assay Kit (Beyotime Biotechnology). The lysates were subjected to 10% sodium dodecyl sulfate–polyacrylamide gel electrophoresis (SDS–PAGE) and transferred to PVDF membranes (MilliporeSigma, Burlington, MA, USA). The membranes were then blocked in 5% non-fat milk powder in PBS-Tween and incubated with primary antibodies against ARHGAP19 (1:500, sc-398428, Santa Cruz Biotechnology, Santa Cruz, CA, USA), E-cadherin (1:2000, A3044, ABclonal Technology, Wuhan, China) overnight at 4 °C. After washing with PBST, the membranes were incubated with horseradish peroxidase (HRP)-conjugated secondary antibodies (1:5000, ABclonal Technology) for 2 h at 37 °C and visualized by chemiluminescent detection using an ECL kit (Beyotime Biotechnology).

### Immunofluorescence

Cells were fixed with 4% paraformaldehyde for 30 min and then permeabilized with 0.3% Triton X -100 (Beyotime Biotechnology) in PBS. After blocking with 4% BSA for 1 h at room temperature, samples were incubated with primary antibody against E-cadherin (1:100, A3044, ABclonal Technology) or OCLN (1:100, Santa Cruz Biotechnology) overnight at 4 °C. An Alexa Fluor® 594-conjugated Goat polyclonal was used as the secondary antibody (1:500, ABclonal Technology). Nuclear staining was performed with DAPI (Beyotime Biotechnology) after which the cells were imaged using a Zeiss LSM780 confocal microscope system (Zeiss).

### Scanning electron microscopy (SEM)

HEC-1-A cells were fixed in 2.5% glutaraldehyde and post-fixed in 1% osmium tetroxide. After washing the samples with PBS, they were dehydrated with a series of incubations in ethanol. Dehydration was continued by incubations in 95% ethanol, followed by absolute ethanol. SEM analysis of the cell surface was performed using an ultra-high-resolution scanning electron microscope (SU8010, Hitachi, Japan) at the Analysis Center of Agrobiology and Environmental Sciences, Zhejiang University (China).

### Dual-luciferase activity assay

A total of 6 × 10^4^ HEK293T cells were seeded in 24-well dishes 24 h before transfection. 500 ng pmirGLO vectors containing wild-type or mutant fragment of *Arhgap19* 3’UTR (Promega, Madison, WI, USA), 50 nM miR-192-5p or scrambled control were co-transfected using Lipofectamine 2000 (Invitrogen). Luciferase activities of cellular extracts were measured 48 h after transfection by using a Dual-Luciferase Reporter Assay System (Promega). Efficiency of transfection was normalized using Renilla luciferase activity. Details for cloning target 3’UTR are as follows: a 589 bp fragment from mouse Arhgap19 3’UTR was amplified with 5’ primer 5’- GAGCATGGAGGTGTGTGATCT-3’ and 3’ primer 5’- GTCTATTCTGCACTGGATCACAG-3’. Mutated constructs were synthesized with the predicted miR-192-5p binding sites modified (wild-type: 5′ TAGGTCA 3′; mutant 5′ GCTTTCA 3′).

### Statistics

All experiments were presented as means ± SDs. Statistical differences between the two groups was analyzed using the two-tailed unpaired Student’s t-test. Comparison among multiple groups was conducted using One-way analysis of variance (ANOVA) followed by Dunnett’s test. Statistical significance was defined as *P* < 0.05.

## Results

### ARHGAP19 is upregulated in receptive mouse uteri and human EECs

**Fig. 1:**
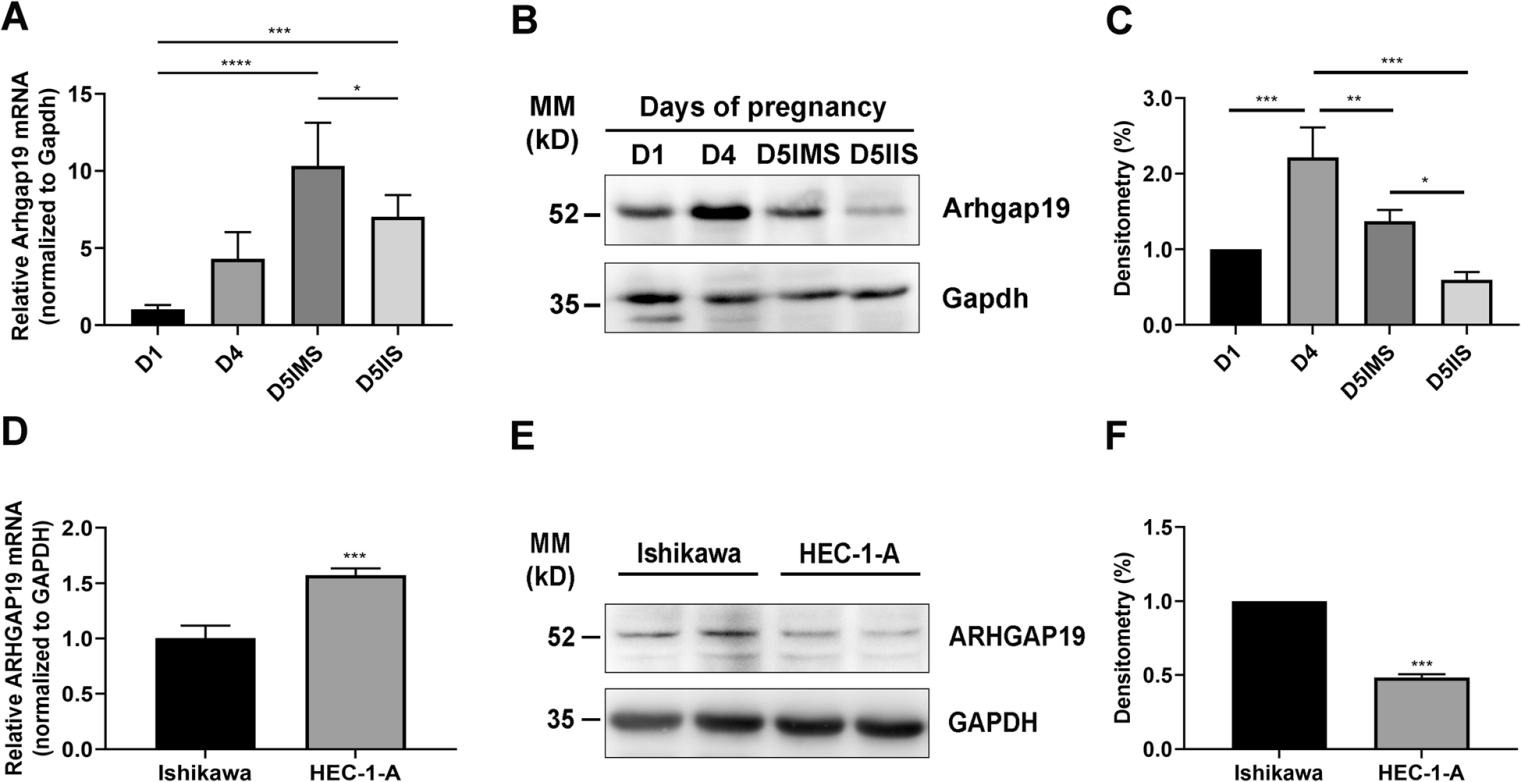
Expression of ARHGAP19 in mouse uteri and human endometrial epithelial cell (EEC) lines. **a** Detection of Arhgap19 mRNA in mouse uteri during early pregnancy. IMS, implantation sites. IIS, inter-implantation sites. **b** Detection of ARHGAP19 protein in mouse uteri during early pregnancy. **c** Densitometry analysis of mouse ARHGAP19 protein presented in **b**. **d** Detection of ARHGAP19 mRNA in receptive (Ishikawa) and non-receptive (HEC-1-A) cell lines. **e** Detection of human ARHGAP19 protein in EEC lines. **f** Densitometry analysis of human ARHGAP19 protein presented in **e**. *P < 0.05, **P < 0.01, ***P < 0.001, ****P < 0.0001

In mice, the uterine sensitivity to implantation can be divided into three phases: pre-receptive (D1-D3 of pregnancy), receptive (D4-D5), and refractory (beyond D5). Implantation takes place at midnight of D4 [13, 14]. We examined the expression pattern of ARHGAP19 during early pregnancy in mice, and found that both ARHGAP19 mRNA and protein were upregulated during the receptive and implantation phase compared to that in the pre-receptive phase. In addition, the expression of ARHGAP19 in the implantation sites was significantly higher than that in the inter-implantation sites (Fig. 1a-c). We further examined the expression of ARHGAP19 in non-receptive and receptive human EEC lines. HEC-1-A cell line displays an intact pattern of intercellular contacts of highly polarized epithelial cells and poor adhesive properties for trophoblast-like cells, which often serves as *in vitro* model for non-receptive uterine epithelium. While the Ishikawa cell line exhibits mild cell polarity and possesses apical adhesiveness to trophoblast-like cells, which makes it a good model of receptive uterine epithelium [15–18]. The detection of ARHGAP19 expression showed that, HEC-1-A cells process higher levels of ARHGAP19 mRNA compared with that in the Ishikawa cells. However, the protein level of ARHGAP19 in HEC-1-A cells was significantly lower than that in the Ishikawa cells (Fig. 1d-f). Taken together, these results indicate that ARHGAP19 is upregulated in the receptive uteri and ECCs.

### ARHGAP19 induces morphological alterations in EECs by regulating the remodeling of junctional complexes and membrane-associated cytoskeleton

Given the relatively high protein level of ARHGAP19 in uterine tissues and cells under receptive conditions, we hypothesized that ARHGAP19 might play a role in establishing receptivity. To test this hypothesis, we increased the level of ARHGAP19 in non-receptive HEC-1-A cells and observed whether the cells could transit to a receptive phenotype.

**Fig. 2:**
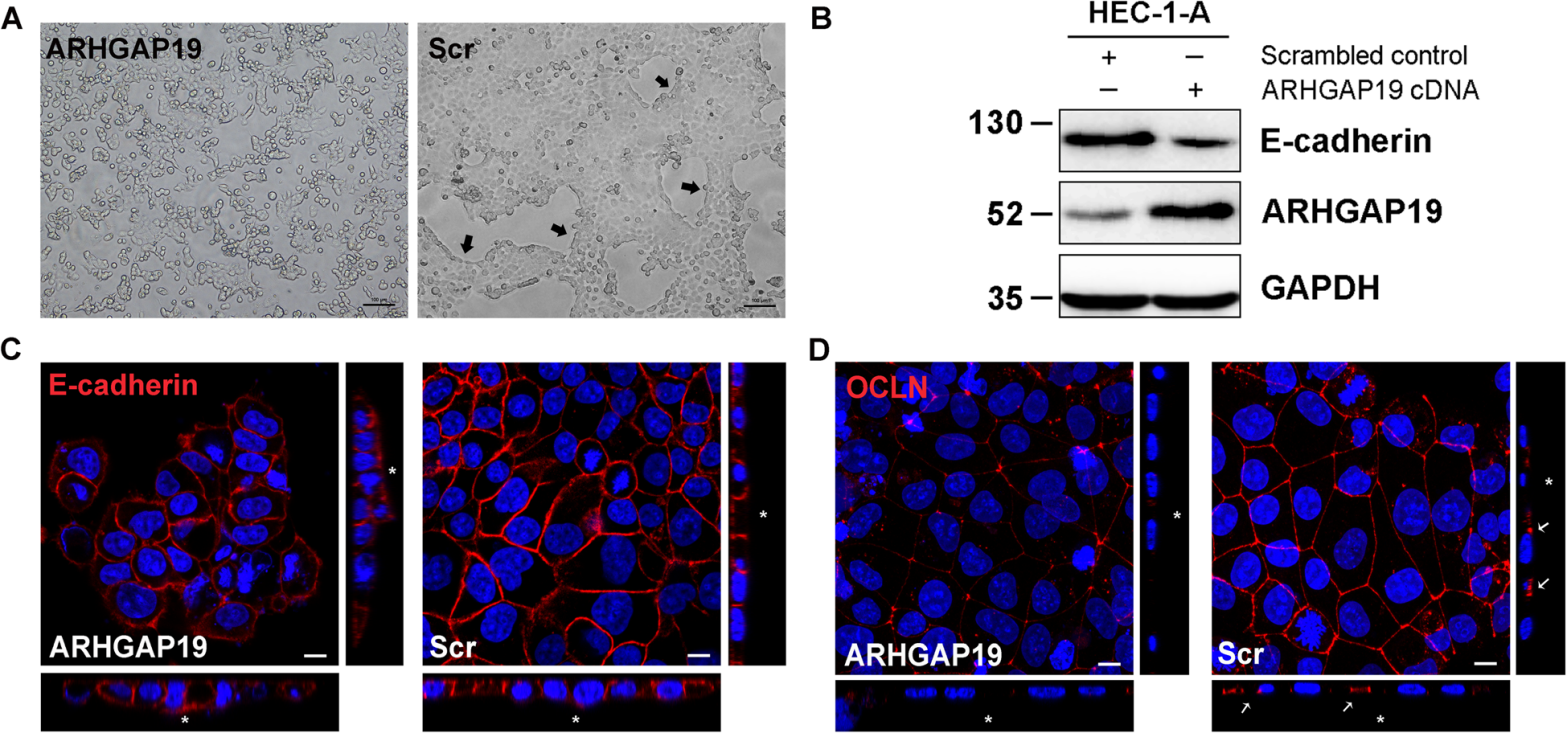
ARHGAP19 overexpression induces the remodeling of junctional complexes. **a** Representative images of HEC-1-A cells transfected with ARHGAP19 or scrambled control (Scr). Scale bar, 100 µm. Arrows point to the boundaries of cell colonies. **b** Detection of E-cadherin expression in HEC-1-A cells after transfection of ARHGAP19. **c** 3D reconstruction of E-cadherin localization by IF in ARHGAP19 overexpressing cells. Scale bar, 10 µm. * represent the apex side of cell. **d** 3D reconstruction of Occludin localization by IF in ARHGAP19 overexpressing cells. Scale bar, 10 µm. * represent the apex side of cell. Arrows pointed to the Occludin in the cell-cell contact

As shown in Fig. 2a, the transfection of ARHGAP19 led to obvious morphological changes in HEC-1-A cells. Normally, HEC-1-A cells form ordered monolayers of cuboidal to columnar cells, as shown in the scrambled control group. Since cells are highly polarized and tightly connected through stable junctions, distinct boundaries can be seen at the edges of the cell colonies. However, ARHGAP19 overexpressing HEC-1-A cells exhibited a more roundish shape and tended to grow in a piled-up fashion. The junctions between cells became weaker and the boundaries of cell colonies were blurred. This phenomenon suggested that ARHGAP19 change cell-to-cell junctions in HEC-1-A cells.

Junctional proteins are key elements that connect cells and give epithelial cells their unique character. Next, we examined the effect of ARHGAP19 on the expression of critical junctional proteins. E-cadherin is the key component that forms adherens junction in epithelial cells. It is expressed in the lateral membrane and maintains cell-cell adhesion in polarized epithelial cells [19, 20]. Overexpression of ARHGAP19 significantly reduced E-cadherin expression in HEC-1-A cells (Fig. 2b). Moreover, immunofluorescence results revealed an altered E-cadherin distribution in the membrane of ARHGAP19 overexpressing cells (Fig. 2c). Unlike the control cells, in which E-cadherin was restricted to the lateral membrane, ARHGAP19 overexpression cells exhibited a random distribution of E-cadherin both apically and laterally. We further examined the expression of Occludin, which plays a role in the formation and regulation of the tight junction, and found that overexpression of ARHGAP19 reduced Occludin expression at the cell-cell contact in HEC-1-A cells (Fig. 2d). These results indicated that ARHGAP19 induces the remodeling of junctional proteins.

**Fig. 3:**
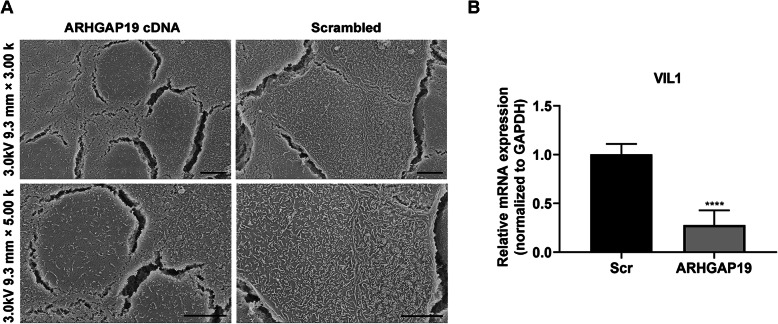
ARHGAP19 overexpression disrupts the formation of microvilli. **a** Representative images of scanning electron microscopy (SEM) in HEC-1-A cells transfected with ARHGAP19. Scale bar, 5 µm. **b** Detection of Villin mRNA in HEC-1-A cells transfected with ARHGAP19. ****P < 0.0001

Besides the remodeling of junctional proteins, cytoskeleton elements, especially the membrane-associated cytoskeleton, experience their own form of transformation during the receptive phase. Of particular note is the retreat of actin-containing microvilli on the apical membrane [21]. We further examined how ARHGAP19 influence the expression pattern of microvilli in HEC-1-A cells. SEM analysis revealed that upregulation ARHGAP19 significantly reduced the number and length of microvilli (Fig. 3a). Besides, ARHGAP19 downregulated the expression of gene that encodes Villin (Vil) (Fig. 3b), an actin-binding protein that promotes the formation of microvilli [22]. Combined with the result that ARHGAP19 alters the configuration of epithelial cells (from columnal shape to a roundish shape), ARHGAP19 was shown to cause the reorganization of cytoskeleton that is similar to the changes occurring during epithelial transformation in establishing receptivity.

### miR-192-5p is an upstream regulator of ARHGAP19

As a member of the RhoGAP family, ARHGAP19 is involved in multiple cytoskeleton-related cellular events. However, little is known about how ARHGAP19 is regulated. We noticed that compared with Ishikawa cells, HEC-1-A cells possess higher level of ARHGAP19 mRNA but lower level of protein, suggesting the possibility of posttranscriptional regulation. MicroRNAs (miRNAs) are small non-coding RNAs that direct posttranscriptional repression of target genes in diverse eukaryotic cells [23]. We thus suspected ARHGAP19 might be regulated by miRNAs.

**Fig. 4:**
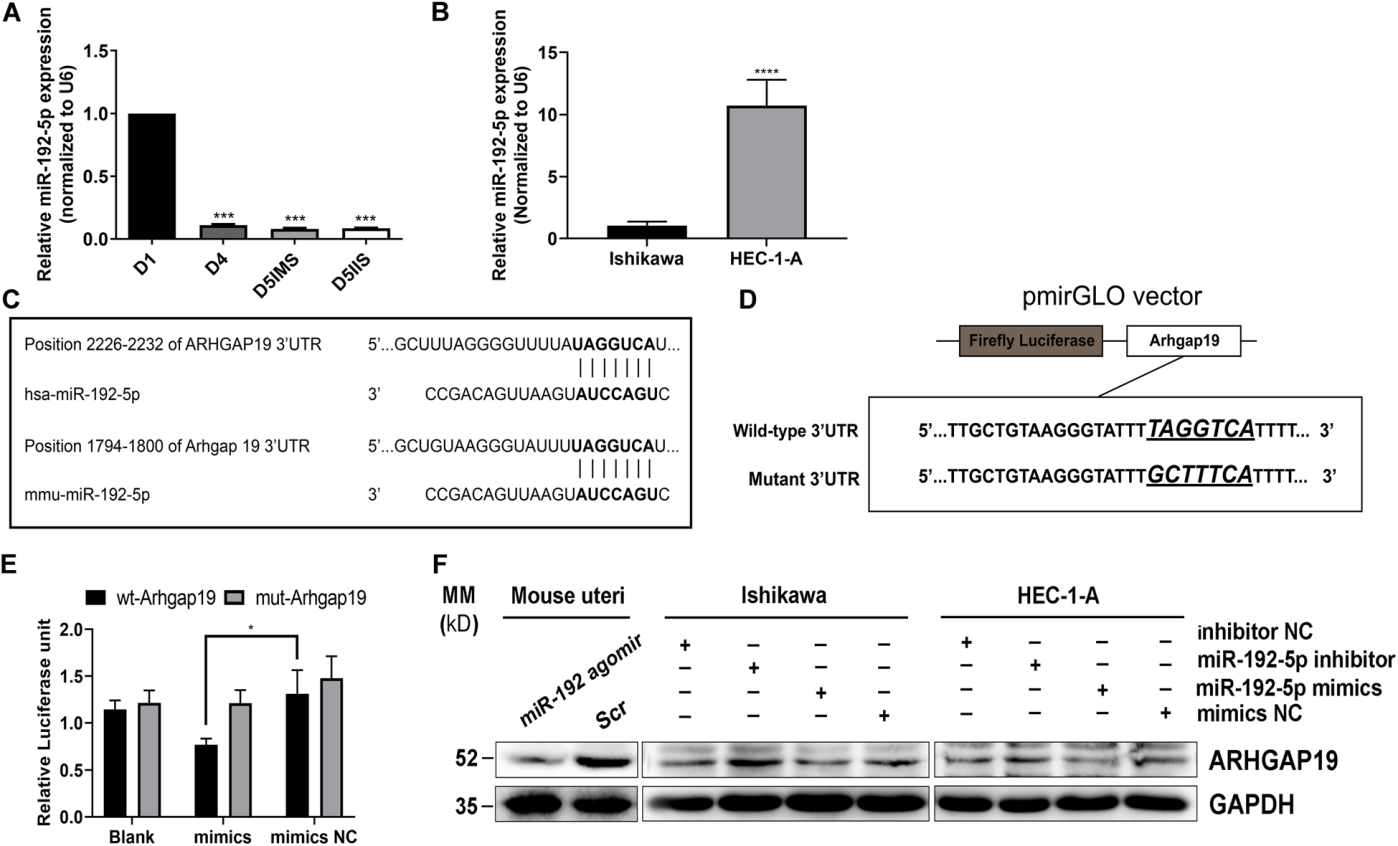
ARHGAP19 is regulated by miR-192-5p. **a** MiR-192-5p expression in mouse uteri during early pregnancy. **b** MiR-192-5p expression in human EEC lines. **c** Putative binding sites of miR-192-5p in the 3’UTR region of ARHGAP19 in mice and humans. **d** A schematic representation of the structure of the wild-type and mutant Arhgap19-3’UTR vector. The mutant vector was generated by mutation of the putative binding sites of miR-192-5p in the 3′UTR region. **e** Relative luciferase activity in HEK293T cells co-transfected with miR-192-5p mimics, scrambled control, and the blank, wild-type (wt) or mutant (mut) Arhgap19 3’UTR vectors. **f** MiR-192-5p regulates ARHGAP19 expression in mouse uteri (left) and human EEC lines (right). *P < 0.05, **P < 0.01, ***P < 0.001, ****P < 0.0001

We previously reported that miR-192-5p functioned in EECs and was downregulated during mouse early pregnancy (Fig. 4a) [24]. We further examined the expression of miR-192-5p in human EEC lines and found that HEC-1-A cells possess a much higher level of miR-192-5p than Ishikawa cells (Fig. 4b). These expression trends of miR-192-5p are all contrary to those of ARHGAP19. More importantly, bioinformatics analysis using three different online platforms (TargetScan, http://www.targetscan.org/vert_72/, miRDB, http://mirdb.org/, miRWalk, http://mirwalk.umm.uni-heidelberg.de/ ) indicated that ARHGAP19 is a potential target for miR-192-5p in both mice and humans (Fig. 4c). To determine the direct target relationship between miR-192-5p and Arhgap19, a dual-luciferase reporter assay was conducted. Luciferase reporters containing either the wild-type or mutant 3’UTR fragment of mouse Arhgap19 were generated and were co-transfected with miR-192-5p mimics or scrambled control sequence in HEK293T cells (Fig. 4d). As shown in Fig. 4e, miR-192-5p mimics led to a significant decrease of 36.5% in the luciferase activity, and mutation of the miR-192-5p binding site abrogated the knockdown effect. We further examined the effect of miR-192-5p on endogenous ARHGAP19 expression in mouse uterus and human EEC lines. As shown in Fig. 4f, the downregulation of ARHGAP19 was found *in vivo* in mouse uterine horns treated with miR-192-5p agomir. Also, in both HEC-1-A and Ishikawa cells, overexpressing miR-192-5p induced a significant reduction of endogenous ARHGAP19, whereas inhibiting miR-192-5p released its expression. These results demonstrate that ARHGAP19 is directly regulated by miR-192-5p.

## Discussion

In preparation for embryo implantation, the endometrial epithelium undergoes remarkable configuration changes, with morphological and functional alterations occurring in the plasma membrane both apically and basolaterally. These changes ultimately led to reduced cell polarity and enhanced apical adhesiveness, facilitating embryo adhesion and invasion [2]. Remodeling of junctional complexes and reorganization of the cytoskeleton play fundamental roles in regulating cell morphogenesis; however, surprisingly, little research has been done on the role of cytoskeletal regulators during the epithelial transformation in early pregnancy. In this study, we unravel the role of a cytoskeleton-associated protein ARHGAP19 in manipulating the morphological transformation of EECs in establishing receptivity.

ARHGAP19 belongs to the RhoGAP family, which promotes the GTPase activity for intrinsic GTP hydrolysis, thus inactivated Rho protein activity [9]. At present, functional studies on ARHGAP19 are quite limited, but based on its expression pattern, that is, highly expressed in fetal tissues and some specific cell types (such as hematopoietic cells), it is speculated that ARHGAP19 may be involved in the regulation of developmental process [25, 26]. Although being less-well characterized, current studies involving ARHGAP19 all suggest that it participates in the regulation of cytoskeleton-mediated morphological changes in cells. For example, ARHGAP19 has been shown to modulate cell elongation and cytokinesis in early mitosis of lymphocytes through regulating RhoA/ROCK signaling [26]. In another study, loss-of-function of ARHGAP19 in epidermal cells was reported to induce actin cytoskeleton reorganization, alter cell polarity, and stimulate adherens junction formation [10]. In the present study, we explored the regulatory role of ARHGAP19 in endometrial epithelial morphology. Despite a relatively low expression of ARHGAP19 in EECs, its expression in receptive and non-receptive cells showed a significant difference. By altering the level of ARHGAP19 in EECs, we found that upregulation of its expression was able to induce the non-receptive cells to acquire a partial receptive phenotype.

The most significant change at the lateral membrane of EECs in establishing receptivity is the remodeling of junction complexes [2]. E-cadherin is a Ca^2+^-dependent cell adhesion molecule expressed in the epithelial cells and plays a critical role in maintaining epithelial integrity and polarity [4]. Downregulation of E-cadherin in EECs allows the adhesion and penetration of trophoblast cells, thus is critical for implantation initiation [27, 28]. Consistent with the previous study [10], we showed that ARHGAP19 induced the remodeling of adherens junction protein through repressing E-cadherin expression. Moreover, we found that ARHGAP19 overexpression led to an atypical distribution of E-cadherin in polarized EECs, i.e., randomly distributed to the whole plasma membrane domains instead of restricted to the lateral domains. The redistribution of E-cadherin may result from an altered interaction with the catenins (i.e. β-catenin and α-catenin) and actin filaments, these alterations subsequently change cell-cell adhesion [29]. Thie et al. reported a random distribution of E-cadherin in another receptive human EEC line RL95-2. These cells usually lack the polarized structure of a typical epithelial cell and exhibit a strong tendency to pile up [15, 18]. Interestingly, in our study, we also observed a piling-up tendency of HEC-1-A cells after ARHGAP19 overexpression, which we speculated might be related to the redistribution of E-cadherin. Besides alterations in adherens junction protein, ARHGAP19 overexpression also reduced the expression of tight junction component Occludin at the cell–cell interface. These results further strengthened the fact that ARHGAP19 induced junctional remodeling that possibly leading to the loss of epithelial polarity.

During the receptive phase, the apical membrane of EECs undergoes remarkable morphological alterations involving the retreat of actin-containing microvilli and the removal of terminal web [21]. These phenomena are found in several species during early pregnancy and are considered as the prerequisite for embryo attachment [2]. In the present study, we found that upregulation of ARHGAP19 was able to cause a decrease in microvilli and resulted in downregulation of the expression of the gene encoding Villin, a calcium-regulated actin-binding protein that modulates the structure and assembly of actin filaments and plays a key role in the morphogenesis of microvilli [22, 30]. These results indicated that ARHGAP19 induces membrane- associated cytoskeletal reorganization, prompting changes in membrane morphology and allowing cells to acquire a receptive-associated phenotype.

In this study, we also found that ARHGAP19 was regulated by miR-192-5p. MiR-192 is located on chromosome 19 in mice and on chromosome 11 in humans. The mature miR-192-5p sequence is identical in mice and humans. Studies on different tissues indicate that miR-192-5p is enriched in the epithelium, and participates in determining or maintaining cell-type-specific characteristics, including epithelial differentiation and ion transportation [31–33]. Our previous study [24] showed that miR-192-5p was significantly down-regulated during the receptive and implantation phase, contrary to the trend of ARHGAP19 expression. Inhibiting miR-192-5p function in HEC-1-A cells suppress E-cadherin expression at cell-cell interface and reduced apical microvilli formation, all of which are similar to overexpression of Arhgap19. This study confirms that ARHGAP19 is a target gene of miR-192-5p. We speculate that the endometrium may release ARHGAP19 expression by downregulating miR-192-5p during early pregnancy, which in turn promotes epithelial morphological transformation.

## Conclusions

In summary, this study investigated the role of the cytoskeleton-associated factor ARHGAP19 in EECs, which may contribute to the transition of epithelial cells from a non-receptive to a receptive state by regulating the remodeling of junctional complexes and membrane-associated cytoskeleton. These results provide a theoretical reference for understanding the mechanisms underlying the establishment of receptivity. However, whether ARHGAP19 exerts its influence on epithelial morphology through its GAP activity remains unknown. Further investigations are required to unveil the down-stream factors for ARHGAP19-mediated regulation.

## Data Availability

All data generated or analyzed in this study are included in this published article.

## Abbreviations

EECs: Endometrial epithelial cells
IIS: Inter-implanation sites
IMS: Implantation sites
RhoGAPs: Rho GTPase-activating proteins
SEM: Scanning electron microscopy
